# Co-Producing an Intervention to Reduce Inappropriate Antibiotic Prescribing Among Dental Practitioners in India

**DOI:** 10.3390/antibiotics14100984

**Published:** 2025-09-30

**Authors:** Aarthi Bhuvaraghan, John Walley, Rebecca King, Vishal R. Aggarwal

**Affiliations:** 1 School of Dentistry, Faculty of Medicine and Health, University of Leeds, The Worsley Building, Clarendon Way, Leeds LS2 9LU, UK; 2 Sree Balaji Dental College and Hospital, Bharat Institute of Higher Education and Research, Chennai 600100, India; 3 Nuffield Centre for International Health and Development, University of Leeds, The Worsley Building, Clarendon Way, Leeds LS2 9LU, UK; j.walley@leeds.ac.uk (J.W.); r.king@leeds.ac.uk (R.K.)

**Keywords:** intervention, medical research council, co-production, stakeholders, India, dental practitioners, primary care, antibiotics, antimicrobial resistance, AMR, antibiotic stewardship, dentistry, education, computer-based, online

## Abstract

**Background:** Inappropriate antibiotic prescribing by dental practitioners is a significant problem in low- and middle-income settings, such as India, where there are no guidelines for dental prescribing. This study aims to report, in a step-by-step process, the co-development of a computer-based stewardship educational intervention with Indian stakeholders to reduce inappropriate antibiotic prescribing by primary care dental practitioners in India. **Methods**: The development process of our intervention was guided by the Medical Research Council framework for developing and evaluating complex interventions. In alignment with the framework’s core elements, a co-production research approach was employed. Engagement with local stakeholders, including primary care dental practitioners, academic dentists, and those from the Indian Dental Association, facilitated the development of a contextually appropriate intervention that was informed by a prior needs assessment (a systematic review and a policy document analysis conducted in India) and evidence from global literature. The intervention was refined through iterative feedback from stakeholders and pre-testing. **Results**: An educational antibiotic stewardship intervention was co-developed in collaboration with stakeholders from Chennai, a major city in southern India. The final intervention comprised three components: 1. A one-page chairside guide summarising common areas of dental antibiotic use for easy reference in clinical settings; 2. A training module based on the chairside guide; and 3. A patient information sheet to facilitate dentists’ communication with patients. The intervention components were designed to be clear, practical, and contextually relevant, with the potential to enhance clinical decision-making and promote evidence-based antibiotic prescribing practices. **Conclusions**: This research paper describes, in a structured manner, how an educational antibiotic stewardship intervention for dental practitioners in India was co-developed by researchers and local stakeholders. Further feasibility testing is required to address uncertainties identified at the conclusion of the development process, including those related to dentists’ perceptions of the intervention, the utility of the intervention tools, and prescription recording.

## 1. Background

Antimicrobial resistance (AMR) is the ability of micro-organisms to resist the effects of medications that were once effective in killing them or inhibiting their growth [1]. In 2019, an estimated 1.27 million deaths worldwide were directly attributable to antimicrobial-resistant infections [2]. This figure is projected to increase to 10 million by 2050, with substantial implications for the global economy [3]. Overuse of antibiotics contributes to the development and spread of resistance [1,4]. In low- and middle-income countries (LMICs) such as India, such overuse occurs in the community setting through unlawful dispensing by informal providers, over-the-counter use by the public, and overprescribing by qualified practitioners. As the largest consumer of antibiotics by volume, India is experiencing an escalating challenge in managing antimicrobial resistance [5,6,7]. By 2050, one-fifth of all AMR deaths will be from India if mitigatory measures are not taken [8].

Dental practitioners account for nearly 10% of all antibiotics prescribed in healthcare [9,10,11]. However, over three-fourths of antibiotics prescribed are found to be inappropriate in high-income countries [12,13]. The problem is particularly acute in India, where antibiotics are not only overprescribed for therapeutic and prophylactic purposes, but also for non-clinical reasons such as patient expectations and pharmaceutical pressure, further contributing to the global burden of AMR [14].

Globally, antibiotic stewardship interventions have been shown to result in increased compliance with guidelines and a reduction in inappropriate antibiotic prescribing among dental practitioners [15]. Approaches such as developing internal guidelines, disseminating existing ones, academic detailing, providing patient leaflets, conducting audits and feedback with peer comparisons, and one-on-one educational discussions, often with education as a central component, have been utilised individually or in combination to improve antibiotic prescribing practices [16,17,18,19,20,21]. In addition to face-to-face interventions, online learning has also been successfully implemented in stewardship efforts around the world [22,23].

However, there are currently no such tailored learning resources available for dentists in India to help them gain an understanding of AMR and guide them on appropriate antibiotic prescribing in dentistry, nor are there established strategies for their delivery [24]. Such limited evidence from context-specific interventions poses a challenge in advocating for the necessity of these measures to decision-makers. Previous research by the authors identified, through a systematic review, the extent of antibiotic misuse in dentistry in India [14]. A policy analysis further demonstrated the deficiency in training and the lack of guidelines and educational resources to mitigate this misuse [24]. These studies highlight a gap in the need to educate Indian dentists to help reduce inappropriate use and optimise their antibiotic prescribing practices.

Whilst stewardship interventions exist globally, caution must be exercised when using evidence from interventions successfully developed and evaluated in high-income (resource) countries (HIC) in the Indian context. While they can potentially lead to improvement in knowledge, the implementation of such evidence into clinical practice in LMICs and low-resource settings is marred by issues around their acceptability, which are brought on by the distinct challenges that practitioners face within their socio-cultural context [25,26]. Practitioners in India receive little benefit for being low antibiotic prescribers, whereas they have strong incentives at multiple levels for potential (over)use—prescribing alleviates fear of losing patients, fulfils their perception of patient expectations, conforms with societal norms, and offers financial incentives from the pharmaceutical industry, all without fear of penalty due to the absence of regulatory monitoring [24,27,28]. Moreover, HICs have well-developed guidelines, and the role of intervention is often to improve practitioner adherence. On the contrary, a lack of dental prescribing guidelines in India means that an intervention would need to improve awareness and understanding of the problem in addition to educating them on the correct usage of antibiotics.

A comprehensive understanding of the local context and resource considerations is essential to ensure the relevance of evidence-based interventions to clinical practice, and thereby their implementation and scale-up [29]. Co-production is a process wherein the participants commit to working together to produce new knowledge. The focus is on bringing together their varying perspectives, which help in shaping research, in this case, the intervention. Very little research on co-production exists in LMIC contexts, partly due to resource constraints, but also likely influenced by unequal power dynamics [30]. Nevertheless, it is crucial for the long-term success of research and ensures sustainability and meaningful impact. This paper systematically describes how an intervention to improve antibiotic prescribing behaviour among dental practitioners was co-produced with dental stakeholders from India.

## 2. Aims and Objectives

The aim of this research was to develop, in partnership with local (Indian) stakeholders, a computer-based intervention package tailored to context and aimed at reducing inappropriate antibiotic prescribing behaviour among primary care dental practitioners in India.

The specific objectives are as follows:

- To engage key stakeholders in India and identify priority topics related to antibiotic prescribing in dental practice.
- To collaboratively design a computer-based educational intervention package that addresses inappropriate antibiotic use, ensuring it is evidence-based, contextually relevant, and feasible for use in Indian primary dental care settings.
- To refine the intervention components through iterative feedback and pre-testing.

## 3. Considering the Core Elements of the Medical Research Council (MRC) Framework

**Figure 1:**
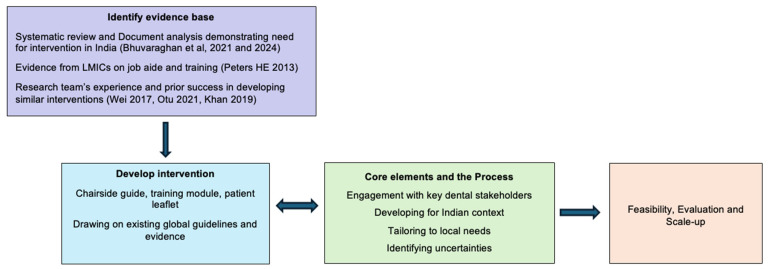
Intervention development as guided by the Medical Research Council (MRC) framework for the development of complex interventions [14,32,33].

Several frameworks are available to guide researchers and stakeholders in developing interventions. In developing the current intervention package, significant consideration was given throughout the process to the following core elements outlined in the Medical Research Council (MRC) framework for the development and evaluation of complex interventions (Figure 1) [31].

### 3.1. Engaging with Stakeholders

Although the researchers themselves had a good understanding of the problem, considered it an increasing priority, and clarified the area of intervention, AMR was not a recognised subject among dental stakeholders in India. Their involvement in our research was therefore not just important but indispensable to incorporate their views and, hence, achieve acceptability among the larger dental community. This initiative engaged a broad range of stakeholders, including researchers, members of the Indian Dental Association (IDA), academic dentists, dental clinicians, and specialists with varying experiences and work settings—all of whom contributed their knowledge and clinical experiences to make this intervention suitable to the context. Accordingly, they contributed to different aspects of the intervention development process at various stages, such as planning, designing content and format, refining, and dissemination (explained in later sections). An initial analytical plan, devised to understand stakeholders and their involvement in the research and development process, is shown in Supplementary Table S1.

Role of the Indian Dental Association: Contact with an executive member of the local branch of the Indian Dental Association (IDA—Madras Branch) was made early on during the development process to provide the IDA with all needed information pertaining to AMR, the need to reduce unnecessary antibiotic prescribing, and our current plans regarding the intervention. The IDA expressed a strong willingness to support the process with respect to

- Helping in identifying/recruiting potential participants;
- Development of resources;
- Endorsing the developed materials;
- Disseminating any developed stewardship resources on their websites;
- Organising stewardship activities, if needed, such as CPD programmes;
- Assistance with future scale-up;
- Bringing the issue to the attention of the dental regulatory authority.

One of the members, a senior clinician, volunteered to be part of our development team—the Technical Working Group (explained later).

### 3.2. The Context

Implementing global interventions in India is challenging due to contextual factors and the nation’s internal diversity. Evidence from HICs shows that guidelines alone, without the presence of education and awareness, have little effect in improving antibiotic prescribing by dental practitioners [16]. Similarly, in India, qualified and unqualified healthcare providers show poor adherence to clinical guidelines, resulting in unnecessary prescribing of medications [34]. Unfortunately, there are neither guidelines nor relevant educational resources on the prescribing of antibiotics in dentistry. Additionally, while developing guidelines is the responsibility of the government and organisations such as the Indian Council for Medical Research (ICMR), the process itself is intricate and demands significant resources [35]. In the absence of local guidelines, Indian practitioners experience difficulty relating to global practices, i.e., those from HICs, making the dissemination of global guidelines less acceptable [35,36]. Therefore, the ‘middle ground’ would be to tailor global evidence and resources to suit the local context, taking into account current practices, norms, beliefs, local resources, and the environment in which they operate. Context determines the content of the intervention and its mechanism of delivery.

### 3.3. The Programme Theory

We used the programme theory to clarify causal assumptions. Based on this, an initial logic model demonstrating the theory of change was created by the research team (Supplementary Figure S1) and refined with the TWG, showing how education, i.e., awareness and knowledge, could improve dentists’ antibiotic prescribing behaviour.

The assumption was that

When

Key stakeholders are engaged to plan, develop, and refine an educational antibiotic stewardship intervention for primary care dentists,

Evidence from global good practices and guidances is considered and combined with prescribing practices from India,

Then

The developed educational intervention is tailored to local needs and context,

Is acceptable to target dentists,

So that

It becomes possible to create awareness about AMR and the importance of rational antibiotic prescribing among dentists,

It improves dental practitioners’ knowledge of and confidence in antibiotic prescribing for common dental conditions, and their communication with patients,

Which, in turn

Reduces inappropriate antibiotic prescribing behaviour of dentists.

### 3.4. Key Uncertainties

Translating complex interventions to clinical practice comes with key uncertainties that need to be clearly identified by researchers and stakeholders. This enables these issues to be either addressed or where this is not possible, to take measures to overcome such uncertainties. In our research, the following key uncertainties were identified: 1. How will effectiveness be measured (tracking improvement in prescribing behaviour)? 2. Will the lack of face-to-face interaction affect outcomes? 3. Will practitioners perceive this training as necessary in their practice? 4. In the absence of guidelines, will practitioners be able to overcome existing prescribing practices, pharmaceutical influence, or patient expectations? 5. Can this intervention be adapted for diverse settings?

Stakeholder engagement and iterative development were therefore essential to address these uncertainties (questions), in addition to feasibility evaluation.

## 4. Methods

### 4.1. Ethical Approval

Ethical approval was obtained in the UK from the Dental Research Ethics Committee (DREC ref: 170121/AB/341) and in India from the Institutional Ethics Committee, Bharath Institute of Higher Education and Research, Chennai (ref: SBDCH/IEC/12B/2021/09). Informed consent was obtained from all participants who were willing to participate, provide data, and allow verbatim record of their discussions/interviews to be published. They were also able to withdraw from the study at any time.

### 4.2. Setting

Chennai, the capital of Tamil Nadu in southern India, is widely regarded as the health capital of the country, thanks to its large healthcare workforce and thriving medical tourism [37]. The Chennai Metropolitan Area, with its leading number of dental colleges and professionals, plays a pivotal role in meeting the oral health needs of its 12 million residents [38]. It is also the site of the host institution and, hence, a pragmatic choice for the research and the intervention development process.

### 4.3. The Intervention Development Process

The development of this intervention package has been guided by the principles of implementation research and prioritised an embedded approach. Implementation research or science encompasses a wide variety of strategies employed to integrate health research evidence and interventions into policy and clinical practice [32,39,40]. An effective way to achieve implementation is through the embedded research and development approach, wherein the intervention is co-produced by researchers and local stakeholders, taking local resources into consideration to ensure its suitability to local needs [29]. This approach is particularly valuable in low-resource settings, where it supports efficient utilisation of available resources. The current intervention draws on evidence synthesis from a systematic review and a policy document analysis conducted in India by the researchers [14,24] (Supplementary Table S2), a review of global guidelines on antibiotic prescribing in dentistry [16,17,18,19,20,21], combined with clinical experience of stakeholders and expert opinions. Table 1 presents the diverse stakeholders engaged in the development of this intervention, along with their respective roles.

Overall, twenty stakeholders were involved at various stages, providing information that shaped the intervention. 

The development process involved the following steps:

1. Development of the initial draft intervention by the Research and Development team.
2. Focus group discussions (FGDs).
3. Enhancing the training module (intervention) based on focus group discussions.
4. The Technical Working Group (TWG) and intervention adaptation through iterative feedback.
5. Pre-testing of the intervention package with dental practitioners.
6. Final refinements with the TWG.

#### 4.3.1. Step 1: Development of the Initial Draft Intervention by the Research and Development Team

Our team of four researchers was involved in developing the draft intervention, drawing on the team’s collective expertise in AMR research, intervention development in LMIC contexts, public health, qualitative and quantitative research, dental and medical prescribing, including clinical experiences in India. At this stage, our objective was to clarify the intervention and how it would be delivered.

##### Choice of the Intervention and Content

Engagement with local stakeholders began before the intervention development process, when members were involved in prior research undertaken by the research team analysing Indian policy documents [24], which helped identify gaps in antibiotic stewardship in India and the need for the current educational intervention. Some of these members continued to be part of the current team, helping in prioritising the content and identifying the format and delivery of the intervention. Informal discussions were held with these previously established contacts from the Madras Branch of the Indian Dental Association and with dental practitioners from Chennai, who offered support with development and implementation. Evidence from LMICs indicates that combining printed materials and job aids with health worker training is more effective than education and training alone [41]. Drawing on this evidence—alongside findings from Indian studies [14,24], global literature [42,43,44,45,46,47], discussions within the research team and with local stakeholders, and input from the Technical Working Group—an intervention package comprising the following components was deemed acceptable and feasible for scale-up within the Indian context.

(i) A chairside antibiotic guide—a one-page illustrated job aid containing dental conditions and procedures encountered in Indian primary care dental settings, for which inappropriate antibiotic prescribing is common, and
(ii) A training module in the form of PowerPoint slides based on this guide.

Members of our research team have developed and successfully evaluated similar interventions in other LMIC settings [33,48,49]; however, this was the first of its kind to be developed with dental stakeholders in India. 

The research team agreed to focus on common dental conditions in India where inappropriate antibiotic prescribing is prevalent, i.e., those that would make the greatest impact, while excluding complex procedures such as culture and sensitivity testing, which are not routinely performed by primary care dentists. For example, a recent systematic review highlighted the use of prophylactic antibiotics (pre- or post-operative) for routine extractions and root canal treatments as key areas of inappropriate prescribing, in addition to non-clinical factors such as pharmaceutical influence, patients’ oral hygiene, habits, and socio-economic status [14]. Notably, these context-specific factors do not find mention in global guidelines developed in HICs [42,43,44,45,46,47], where dentists are typically trained to avoid routine prophylaxis for dental procedures. Moreover, dentists from HICs rarely encounter the socio-cultural factors influencing those in LMICs such as India. Similarly, communicating with patients was identified as another important area to be addressed in the training module.

The chairside antibiotic guide therefore comprised common areas of antibiotic use and misuse in the dental outpatient setting, along with prescribing indications and antibiotic regimens. A pictorial representation of the tooth and common diseases of the dental hard and soft tissues was added for clarity. The chairside guide was designed as a quick-reference tool for dental practitioners, and guidance for its use was included in the training module.

The initial draft of the training module included four main sections: 1. Awareness about AMR, 2. Patient assessment to justify antibiotic use, 3. Importance of dental procedures with information on global evidence and guidelines, and 4. Patient communication. Case studies and exercises were included for reinforcement. A questionnaire was also developed for practitioners to complete before and after the training to assess changes in scores (knowledge). It comprised questions related to antimicrobial resistance and antibiotics, case-based scenarios derived from the clinical practice experiences of dentists, highlighting those where antibiotics were commonly, but inappropriately, prescribed.

##### Format and Delivery

The training module (intervention) was designed to be in PowerPoint format, with slides containing learning resources in various sections as mentioned above, videos, and self-assessment exercises. Participants were to read the contents of the module on their devices and undertake a pre-/post-quiz. The goal was to eventually convert this material into an online continuing professional development (CPD)-type resource, to be accessed free of cost by dental practitioners in India. The format was in such a way as to maximise feasibility, so a duration of about 40–60 min was planned, so that practitioners could complete the training at their convenience at home or in their clinics between patients. At the end of the training, participants were to take with them a laminated copy of the job aid to use in their practices.

Given the vast size of the country and the large number of practicing dental clinicians, delivering this intervention through face-to-face methods, such as seminars or workshops, was an enormous undertaking with significant financial implications, especially in remote and rural settings. An online mode of delivery was considered to offer broader reach and was deemed suitable, cost-effective, and feasible, with significant potential for scalability.

##### Critical Knowledge Gap to Be Addressed

To understand if our intervention was indeed effective in reducing antibiotic prescribing among dental practitioners, it was essential to examine prescription numbers and indications. However, there was some uncertainty regarding whether and how dental practitioners in primary care (private) practices recorded and/or retrieved their prescription data. Additionally, there was no existing literature addressing how dentists in India managed their records. While evidence from medical practitioners indicates that paper records were the norm in most primary care settings [50], it was necessary for us to understand dentists’ record-keeping practices, especially their prescription recording, to effectively measure the intervention’s impact and facilitate future audit and feedback processes. An exploratory study in the form of a focus group discussion was deemed essential to gain insight into this.

#### 4.3.2. Step 2: The Focus Group Discussions (FGDs)

##### Purpose

The purpose of the focus group discussions (FGDs) was twofold: 1. To obtain insights into how general dental practitioners maintained a record of their prescriptions, and 2. To refine the initial draft of the chairside antibiotic guide (job aid) developed by the researchers to inform any modifications to the training module.

##### Sampling

Eight primary care dentists (male = 6; female = 2) practising in different settings in Chennai participated in this focus group discussion. While the IDA helped in identifying potential participants, their selection into the focus group was based on purposive sampling. No incentives were provided for participation in this research, and all candidates were interested volunteers. The focus group (FCG) comprised three academic dentists and five full-time dental clinicians. All of them owned their private clinics, practised in different settings, and had variable levels of experience in clinical practice.

##### Methods

All participants were convened for the focus group discussion at a mutually agreed-upon venue (a conference hall), confirmed via telephone at least one week in advance.

Participants provided informed consent for their discussions to be recorded and transcribed and for their verbatim transcripts to be published. On the day of the discussion, the lead researcher (AB) explained to the participants the purpose of the research and what was expected of them. Candidates were interested and volunteered to provide feedback on the job aid as well as their inputs for this research. A senior clinician and researcher (BT), who helped with identifying participants, served as a moderator. Participants were first asked about their current practice of prescription writing and record-keeping and how this could be incorporated into routine practice. The topic guide for the focus group is attached in Supplementary Figure S2. Subsequently, they were each given a printed copy of the job aid to provide feedback on it. The focus group discussion lasted approximately 2 h. 

##### Analysis

The discussions were audio recorded, transcribed verbatim, and reviewed thoroughly before analysis. Further review of transcripts helped identify themes. Data analysis was performed manually without the use of software, as the volume and complexity of the data were manageable. Themes and codes were developed by AB and reviewed and agreed upon by other researchers (RK, VA).

##### Results

The eight participants ranged in age from 26 years to 48 years and provided insights on prescription recording in dental practice, as well as feedback on chairside antibiotic guides, in addition to their prescribing practices and beliefs.

Prescription recording: 

Three major themes emerged on prescription recording.

Theme 1: Recording prescriptions not a norm in primary care dental practice

It was found that seven out of the eight dentists did not routinely record/save copy of prescriptions, although most (n = 6) of them maintained a case sheet where they kept a record of other patient details that they believed to be important for care. The reasons cited included habitual practice, a lack of perceived benefits, the absence of monitoring or audits by regulatory bodies, not encountering patient-related issues due to current practice, as well as due to the complexity of methods and time consumption.

*“* *There is no big use to me recording that* *(prescription)* *data now* *… I* *f I was affected… in the sense some* *two* *of my patients sued me and I had to go to the court of law, then I will automatically start recording even without anyone’s advice.* *” FG 5*

*“If the patient is allergic to a certain medication, I mention that in the case sheet. But I do not routinely record what I prescribe.” FG 8*

Theme 2: Motivating dentists to adopt prescription recording in dental practice

Participants believed that making dental practitioners adopt recording practices could only be achieved through a combination of regulatory enforcement and raising awareness about its importance.

*“If we want them to keep a record, we need to tell first them what will happen if they do not do it. It can be a moral or legal reason.” FG 2*

Theme 3: Multiple ways to record prescriptions in dental primary care 

Additionally, participants provided insights on various ways in which to record prescriptions—both paper-based and digital. Practitioners exhibited varied preferences: While some considered case sheets and carbon-copy prescription pads to be simple and more practical, others regarded digital and computer-based modalities, including mobile applications, as the future of clinical documentation. However, many dentists had reservations about using software due to concerns about data safety and confidentiality.

*“We were also at that time not convinced about software data safety and cloud storage. But in future, things may change.” FG 3*

The key themes and quotes on prescription recording are presented in Supplementary Table S3.

Feedback on antibiotic guide (job aid):

When presented with the one-page job aid, all focus group members expressed satisfaction with the format and idea of the document.

*“I think this is enough, nothing more is required. This will work.” FG 1*

The following themes on antibiotic beliefs and practices emerged from discussions on the job aid. 

Theme 1: Antibiotic routinely prescribed in dental practice

On examining the job aid in detail, dentists were surprised about the lack of indication for antibiotics for most dental diseases and procedures for which they usually prescribed antibiotics, as well as in the choice of the antibiotic (Supplementary Table S4).

*“No antibiotics for extractions?!” FG 3*

They admitted prescribing for a wide range of dental conditions, driven by both clinical and non-clinical factors.

*“You cannot skip antibiotics for abscesses.” FG 4*

*“Instruments we use are not 100% sterile. We only use normal gloves that are not sterile for surgical procedures.” FG 7*

Theme 2: Poor understanding about AMR and antibiotics

Antibiotics were considered a ‘quick fix’ for all dental issues. There was little understanding among dental practitioners about their role in antimicrobial resistance or stewardship.

*“Whatever may happen, antibiotics will take care of it.” FG 2*

*“My personal opinion is that AMR is never going to be caused by dentists in this lifetime… We use very limited number of antibiotics, mostly amoxy (amoxicillin). How can just amoxy lead to AMR?” FG 5*

Participants used a wide variety of antibiotics, with prescribing patterns shifting in response to individual preferences and beliefs, clinical judgment, and situational demands. Amoxicillin with clavulanic acid was favoured by most participants based on their belief that plain amoxicillin lacked effectiveness. Use of parenteral antibiotics, second line, and combination antibiotics was reported.

*“I don’t think amoxy (amoxicillin) works. In case I must give antibiotics, I give only Amoxiclav (amoxicillin with clavulanic acid).” FG 1*

*“After COVID, azithromycin has become resistant. We are using more of Clindamycin.” FG 4*

Theme 3: Need for awareness and dentist-patient communication

Practitioners believed that it was important to create awareness among dental practitioners about AMR, so that they prescribe antibiotics judiciously.

*“Ideally, we need to go to every clinic and talk to dentists.” FG 1*

*“The first reason why dentists have poor practice is because of prescribing antibiotics. Patients don’t go for treatment at all.” FG 2*

It was generally reported that patients had little knowledge of antibiotics, and effective dentist–patient communication was considered important to create awareness among patients.

*“There is a tendency from the patient side to ask for a prescription because they have been treated that way for generations. It is our bounded duty to tell them.” (Senior clinician FG 1)*

*“Many patients do NOT know what antibiotics are.” FG 8*

However, participants who were early in their careers reported difficulties in establishing trust with patients, an issue they felt senior practitioners typically did not encounter. These younger clinicians expressed a fear of losing patients and emphasised the importance of earning patients’ goodwill as a foundation for establishing a successful practice.

*“These dentists (pointing to senior clinicians) are already established. The problem is… patients listen to doctors because you are already an established practice. That’s not the case with us.” FG 5*

*“If you have earned enough to sustain, you can take a stand… But it takes a bit of time.” FG 3*

Theme 4: Government regulations

Practitioners felt that the government needs to enforce stricter regulations on OTC antibiotic sales. They also highlighted concerns about the influence of pharmaceutical sales representatives in promoting antibiotics, suggesting that such endorsements should be restricted or banned to ensure rational prescribing.

*“Many young dentists… they see lot of (sales) representatives. These ‘rep’ meetings should be banned.” FG 1*

*“During COVID time, they were able to successfully implement ‘No OTC sale’. That means it is possible, isn’t it?” FG 5*

It was evident from this small exploratory study that recording (antibiotic) prescriptions was not a routine practice among primary care (private) dental practitioners in India—a crucial finding that needed to be addressed in the training module. In addition, while not part of our objectives, we gained valuable information about dentists’ antibiotic beliefs, practices, and challenges they faced, which triangulated with findings from our systematic review [14]. These findings were integrated to enhance the intervention.

#### 4.3.3. Step 3: Enhancing the Training Module (Intervention) Based on Focus Group Discussions

Based on evidence from this formative study, a separate section on record-keeping was added to the training module, incorporating information on prescription recording in the primary care dental setting and the ideas put forth by the FCG members to motivate dentists to adopt the same. The section included the importance of recording prescription data in clinical practice and suggested multiple ways (manual and digital) by which this could be accomplished in primary care. Supplementary Table S3 enlists the various ways suggested by participants by which prescription data could be recorded by dentists. Furthermore, a short audio of a dental practitioner narrating his personal experience on how recording prescription data helped him protect himself from a potential lawsuit was added. 

The awareness section was also enhanced to include information on how AMR spreads in the community and the role of dentists in stewardship. A further ‘patient information sheet’ was added to enable dentists to communicate about AMR with their patients. It included information on antibiotics, along with a clear statement that dental procedures, not antibiotics, are responsible for curing toothaches and gum diseases, and incorporated the Indian government’s advisory on the use of antibiotics (in English and the local language Tamil). These sections and the audio were then reviewed by the TWG and subsequently underwent pre-testing for suitability and understandability.

#### 4.3.4. Step 4: The Technical Working Group (TWG) and Intervention Adaptation Through Iterative Feedback

##### Purpose

The TWG was created to review the accuracy and relevance of the content of the training module. In addition to providing initial inputs, they were also required to thoroughly review and provide subsequent feedback and modifications (iterative refinement) to the intervention components.

##### Selection and Composition of the TWG

The TWG comprised four experienced dental practitioners (with a minimum of 15 years of clinical practice in primary care dentistry) practicing in diverse settings in Chennai, along with the lead researcher (AB). As antimicrobial stewardship in dentistry was still in its early stages, identifying experts or researchers working on developing similar interventions in primary care or actively engaged in dental antibiotic stewardship at the grassroots level was challenging, and a more practical approach was taken in selecting the TWG team. Prior contacts in India and within the Indian Dental Association helped narrow down choices for the TWG team. Clinicians were selected based on their relative familiarity with global antibiotic prescribing guidelines, their alignment with the research team’s goals for AMS, and their willingness to contribute to contextualising the content and addressing uncertainties. Participants had substantial experience with common clinical situations in dental practice and were willing to dedicate time to critically reviewing and editing the intervention materials, while providing valuable insights into the content, format, and delivery mechanisms.

Of the five dental clinicians, two were academic dentists who also ran their clinical practices—one was part of the Indian Dental Association’s executive committee, and another contributed to drafting the dental curriculum. The other two were full-time dental clinicians and antibiotic champions, while the fifth practitioner contributed experiences from India and the UK while coordinating the TWG and research team.

##### Methods

The completed version of the intervention package was created following inputs from the FGD. Each of the TWG members was first contacted individually by phone at the beginning of the development process to explain the purpose of the research and their role in developing and adapting the intervention. All members were motivated and agreed to review several iterations of the resource.

In order to obtain a thorough review of the content, we followed a pragmatic iterative approach that was agreed upon with all TWG members. An e-mail of the training module (containing the job aid and patient information sheet) was initially sent to each of the participants, who were given 2 weeks’ time to review the resources slide by slide at their convenience and e-mail back their comments. A subsequent e-mail was sent to them after each of their comments was addressed. Where feedback was unclear or there was conflict, a note (comment) was made by the researcher on the specific slide. Gathering all members at one place at the same time proved to be a challenge within our timeframe. Therefore, a week after sending the second set of e-mails, the lead researcher (AB) met with each of the TWG members individually at their workplace after a prior-arranged appointment to discuss and confirm each slide. This face-to-face meeting also allowed resolution of conflicting ideas, both within the TWG and between TWG member(s) and the researcher.

##### Results

All TWG members were happy with the final content and format of the job aid. The training module was modified to incorporate the following suggestions:

Video on AMR and statistics from India was added,

Regulations regarding record-keeping (Government of India/Dental Council of India) was included,

Patient communication section was refined, and a case scenario was included,

References to global guidelines were added.

Further discussions were conducted within the research team (AB, JW, VA) as part of informed decision-making, ensuring that findings from the TWG were effectively communicated and aligned with global best practices and local evidence.

#### 4.3.5. Step 5: Pre-Testing of Intervention Package with Dental Practitioners

##### Purpose

The pre-testing process was conducted to ensure readability and understandability, to assess approximate duration of the training when presented to an average dental practitioner, to validate intervention components, and to provide a further opportunity to refine the tools. It also ensured that the pictures, videos, and other interactive materials conveyed the intended information.

##### Sample

A sample of four dental practitioners (male = 2; female = 2), selected through convenience sampling, pre-tested the intervention tools.

##### Methods

The practitioners were individually approached face-to face by the researcher (AB) to review the intervention tools (the training module including the job aid, the patient information sheet, and the questionnaire) in the same way as online CPD material is undertaken by dentists. After completing the questionnaire, each dentist reviewed the training module slides on a laptop computer and provided feedback on the clarity and comprehensibility of the content, while the researcher documented the suggestions offered. Following this, the dentist completed the same questionnaire once again (post-test), helping them self-evaluate if the intervention (module) improved their knowledge. Participants also suggested modifications for clarity, or advised the addition or removal of content; for example, removal of a clinical scenario not encountered commonly in practice, or modification of words to align with those terminologies (colloquial terms) used by Indian dentists, etc. Consensus with other participant dentists was verified during subsequent pre-testing.

##### Results

As this process was conducted to test for clarity, understandability, and acceptability of content only, no analysis was involved, and the feedback (descriptive data) recorded resulted in the refinement of intervention components. The main suggestions included modifications to the format or words to make the slides and questionnaire more understandable for the target dentists with whom it will be piloted and eventually implemented. Changes included:

In the questionnaire—changes to antibiotic type/duration based on common use, adding common brand names for analgesics and antibiotics; refinements to case scenario to provide clarity (e.g., adding information on radiographs, patient occlusion, and consulting the medical practitioner); and case scenarios were edited as suggested.

In the module—altering font sizes; splitting slides with too much information; clarifying terminologies such as ‘stewardship’; the addition of a separate slide to explain AWaRe, NLEM, and FDCs; and including ‘catchy’ phrases to be effective.

**Figure 2:**
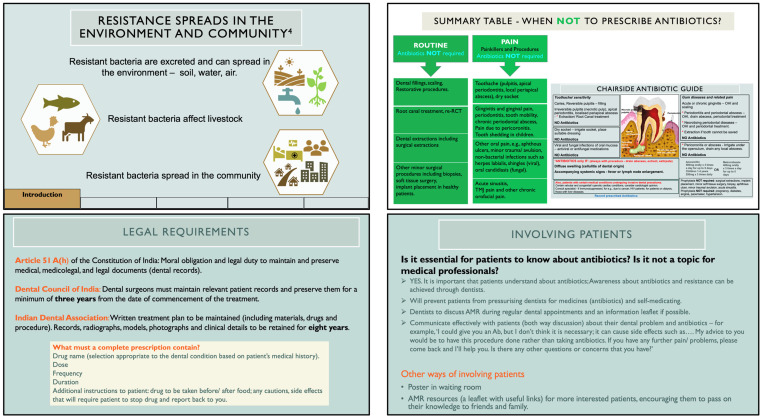
Sample slides from the training module including chairside antibiotic guide.

Figure 2 shows sample slides from the module at the end of the pre-testing stage.

#### 4.3.6. Step 6: Final Refinements with the TWG

The final intervention package that was pre-tested and refined was emailed to each of the TWG members to ensure they were happy with the changes made. A joint meeting was convened online (Zoom call) to address specific issues of concern and ensure the intervention was ready for the next stage. No formal consensus exercises were undertaken. The researcher (AB) presented the refinements made following pre-testing with dentists, all of which were reviewed and approved by the Technical Working Group (TWG) members. Supplementary Table S5 outlines the contents of the training module.

**Figure 3:**
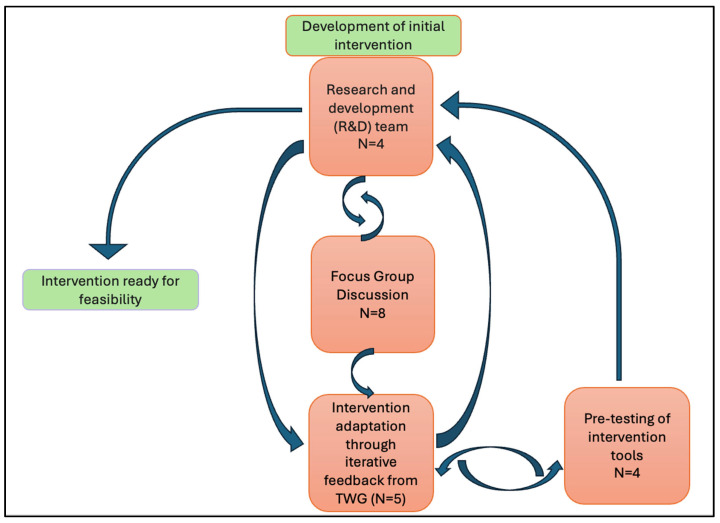
Flow chart of the development process.

A further discussion with the Research and Development team was conducted to reaffirm that the intervention tools were ready to be piloted. Figure 3 illustrates the steps involved and the feedback loops.

The intervention is described using the TiDIeR checklist (Supplementary Table S6). The GUIDED (guidance for reporting intervention development studies in health research) checklist has been used to report the development process (Supplementary Table S7) [51].

## 5. Discussion

This paper describes the co-production of a computer-based educational intervention aimed at improving antibiotic prescribing practices among primary care dentists in India. The development followed an iterative, stakeholder-driven approach based on best available evidence [14,24]. To our knowledge, this is the first such intervention to be developed in India for dental practitioners. The use of TIDieR and GUIDED checklists was instrumental in reporting the process transparently. Clear documentation of the context and development process enables practitioners and policymakers to determine the intervention’s relevance and guide its adaptation to diverse settings.

Engaging a diverse group of stakeholders, i.e., clinicians, academics, and those from the national dental association, helped prioritise intervention components and ensure that the intervention addressed both clinical and socio-cultural influences on prescribing. The iterative feedback loops strengthened content relevance and feasibility [31]. India’s National Action Plan (NAP) highlights the need to develop training resources for healthcare personnel, including dentists, and to secure ratification by professional associations as part of the ongoing efforts to implement antimicrobial stewardship across the country [52]. The current resource has been developed in alignment with the NAP’s goals, with the involvement of the Indian Dental Association from the outset, whose endorsement will lend credibility and facilitate scale-up.

While clinical guidelines informed the core content, several adaptations were made to reflect local prescribing behaviours and systemic constraints. For example, culture and sensitivity testing was excluded due to its limited feasibility in primary care. The emphasis was on practicality over perfection to support effective implementation by dentists in real-world settings [29].

The exploratory study (focus group discussions) revealed important insights about prescription recording practices in primary care dentistry. Two critical findings emerged from this study that helped shape the intervention: a lack of electronic recording system in primary care dentistry and a failure to routinely record prescription data by dental practitioners. Primary care dentists are required by law to preserve all patient records, including prescription data, for a period of 3 years [53]. However, Electronic Health Records (EHRs) are not commonly used in dental practices, where prescriptions are often handed to patients without a copy being retained [50]. In addition, dental records and rational prescribing are not only poorly monitored but also not part of any accreditation process [54]. While the Indian government put forth standards for EHRs [55], there is poor adoption due to costs, time constraints, usability issues, and concerns about data safety [50]. It was therefore essential to address this issue in our training module.

Online educational programmes have been shown to improve knowledge among dental students [23] and prescribing practices among clinicians [22]. However, a recent study from India showed little impact of an online module on dental students’ knowledge [56]. This could be attributed to a lack of emphasis on AMR in their training and the dependence on trainers/instructors for prescribing [24,56]. Additionally, the WHO’s MOOC (Massive Open Online Course) was used in this study, which could have been less acceptable in the Indian setting. In contrast, the current targeted educational intervention was developed in collaboration with local stakeholders, acknowledging the vital role of co-production and embedded development in facilitating implementation [29].

In low- and middle-income country (LMIC) settings, individual prescribing behaviour is shaped by a complex interplay of broader social and environmental factors [25,57]. As such, training interventions must address influences beyond the individual, including patient expectations, peer and pharmaceutical ‘sales representative’ pressures, suboptimal infection control practices, and the absence of clear clinical guidelines. While behaviour change models offer valuable insights, relying solely on these frameworks may be insufficient if systemic barriers, such as limited resources or weak regulatory enforcement, impede prescribers from acting on their intentions [25,26,58]. Conversely, focusing exclusively on ecological or structural factors may also prove inadequate if prescribers lack the requisite knowledge, skills, or intrinsic motivation to change their behaviour. This intervention, unlike many interventions developed in high-income countries [17,19,59], was co-produced specifically for the LMIC setting, addressing gaps that are often overlooked in global guidelines.

Another key strength of the intervention is its online accessibility, which provides flexible and convenient access irrespective of geographic location. This digital format overcomes traditional barriers such as travel and resource limitations, enabling dental practitioners in remote areas to engage with the content [60,61]. Moreover, online delivery facilitates timely updates and scalability, allowing the intervention to be adapted and expanded as necessary [61]. Given the widespread internet connectivity across the country, including rural areas, online learning has the potential to reach participants from diverse demographic groups and practice settings, thereby ensuring equitable access.

At this stage, efforts were made to address key uncertainties identified at the outset of the development process—such as prescription recording, which was incorporated into the training module, and the intervention’s acceptability and potential for future implementation, which were explored through co-production and engagement with the IDA. Further research is needed to evaluate its effectiveness and address residual uncertainties such as prescription recording, perceptions about the intervention, utility of intervention tools, and adaptability to diverse contexts.

## 6. Limitations

One limitation is the limited expertise in dental antibiotic stewardship within the country, as this field is still in its early stages. By involving the professional association (IDA), every effort was made to select the most suitable candidates for the development and refinement process. Additionally, the stakeholder sample was drawn from a city in southern India, which may restrict the generalisability of our findings given the country’s diverse sociocultural, linguistic, economic, and educational landscape. Nevertheless, this document is designed to be dynamic and adaptable, allowing for tailoring to various local contexts.

The use of one focus group to understand record-keeping practices of primary care dentists could be considered a potential limitation. While one focus group is not a representative of all dental practitioners, considering our narrow objective and available time, we believe this was sufficient to adequately address our research objective and obtain valuable insights about prescription recording.

The intervention was designed for dental practitioners to enable them to reduce antibiotic misuse, and while patients are the end receivers of antibiotics, they were not directly involved in the development process. However, aspects of communication were addressed in the module to enable dentists to reduce antibiotics prescribed on account of perceived patient expectations. Feasibility testing of the intervention will determine if and what further research involving patients is required to explore their perspectives and influence on antibiotic prescribing by dentists in India.

## 7. Conclusions

This paper describes in a transparent and structured manner the systematic co-development of an antibiotic stewardship intervention comprising a chairside antibiotic guide, a training module, and a patient information sheet, for dental practitioners in India. By engaging key stakeholders, including professional associations and frontline dentists, the intervention was designed to be contextually relevant, practical, and accessible. The process addressed key gaps in local antibiotic prescribing behaviours and integrated principles of co-production to enhance acceptability and feasibility. The intervention offers a scalable model with the potential to inform national training initiatives and support rational antibiotic use in dentistry. Future work should focus on evaluating the feasibility and acceptability of the intervention and assessing its effectiveness in real-world clinical settings.

## Figures and Tables

**Table 1:** Stakeholders and their contributions towards the intervention development process.

Key Actors/ Stakeholder Groups Involved in Development	Composition Number (n)= Male (m)/Female (f)	Background and Expertise Brought to This Research	Contribution/Role in Development
The Indian Dental Association	n/a	n/a	Recruitment of participants—FCG, pre-testing and subsequent piloting, resource development, endorsement, dissemination, lobbying with the dental regulatory body (Dental Council/NDC)
Researchers (Research and Development Team) (AB, RK, JW, VA)	n = 4 (m = 2; f = 2)	AB—Dental practitioner and specialist in oral medicine. Lived and has work experience in India and the UK. Understands the Indian context and speaks the local language (Tamil). RK—Social scientist and anthropologist. Expertise in qualitative research, intervention development, and participatory approaches in LMIC contexts, and AMR research. JW—Medical practitioner and specialist in public health. Expertise in AMR research, intervention development in LMIC contexts, primary care health service delivery. Prior success in developing and evaluating online educational resources to improve health behaviours. VA—Dental practitioner and specialist in dental public health. Expertise in epidemiology, quantitative research, management of acute and chronic orofacial pain.	Conducted a needs assessment prior to intervention development to determine the need for and the type of intervention. Developed the initial intervention and refined it based on iterative feedback from the TWG, FCG, and pre-testing.
Focus Group	n = 8 (m = 6; f = 2)	Four Academic dental practitioners Four Full-time dental clinicians All members had their own private practices in urban or peri-urban settings within Chennai and had varying levels of clinical experience, ranging from 3 years to 20 years.	Provided inputs on the chairside antibiotic guide (job aid) Provided information on record-keeping and various ways of prescription recording/retrieval in primary care. Helped triangulate findings (from systematic review and document analysis) regarding antibiotic prescribing practices and AMR awareness.
Technical Working Group (TWG)	n = 5 (including lead researcher AB) (m = 3; f = 2)	Dental practitioners from India with over 15 years of clinical experience. AMK—Academic dental practitioner and researcher familiar with global antibiotic guidelines; involved in dental curricular development. KGS—Academic dental practitioner, holds an executive post in the Indian Dental Association. BJK—Full-time clinician, antibiotic champion, holds international (JCI) accreditation in the clinic for quality and patient safety. SJ—Experienced full-time clinician and antibiotic champion. AB—Dental practitioner and academic researcher, co-ordination between R&D team and TWG.	Critically reviewed the accuracy of the content and provided multiple iterative refinements. Provided inputs on the format of intervention. Made final refinements and agreed on the intervention after pre-testing.
Pre-testing	n = 4 (m = 2; f = 2)	Two academic dental practitioners and two full-time practitioners, with varying clinical experience, practising in urban or peri-urban settings within Chennai.	Critically reviewed the content for readability, understandability, and duration of content of the module and the questionnaire for regular dental practitioners.

## Data Availability

Data will be made available upon reasonable request to the corresponding author.

## Supplementary Materials

The following supporting information can be downloaded at: https://www.mdpi.com/article/10.3390/antibiotics14100984/s1, Table S1: Structured brainstorming of the research and development process. Table S2: Understanding the problem of Inappropriate Antibiotic prescribing in dentistry in India - the 6SQuID approach. Table S3: Themes on recording prescription data in primary care. Table S4: Themes on Chairside antibiotic guide. Table S5: Outline of the final intervention (training module). Table S6: The TIDieR (Template for Intervention Description and Replication) Checklist. Table S7: GUIDED (Guidance for reporting Intervention Development studies in health research) Checklist. Figure S1: Draft Logic model of dental antibiotic stewardship intervention. Figure S2: Topic guide for Focus Group Discussions.

## Abbreviations

AMR	Antimicrobial Resistance
AMS	Antimicrobial Stewardship
MRC	Medical Research Council
CPD	Continuing Professional Development
EHR	Electronic Health Records
FCG	Focus Group
FGD	Focus Group Discussions
HIC	High-Income Countries
IDA	Indian Dental Association
LMICs	Low- and Middle-Income Countries
R&D	Research and Development Team
TWG	Technical Working Group
