# Supplementary material for: Co-Producing an Intervention to Reduce Inappropriate Antibiotic Prescribing Among Dental Practitioners in India
Source: Antibiotics (Basel). 2025 Sep 30;14(10):984. doi: 10.3390/antibiotics14100984 (PMC12562034; doi:10.3390/antibiotics14100984)
Supplement: Supplementary file 1 [file antibiotics-14-00984-s001.zip › antibiotics-3848856-supplementary.pdf]

Supplementary files: Co-Developing an Intervention to Reduce Inappropriate Antibiotic Prescribing Among Dental Practitioners in India

Table S1: Structured brainstorming of the research and development process

Development process		Research process
**Who**	**What**	**What**
External panel (Research and Development Team) AB, VA, RK, JW	One-to-two-page chairside guide<br>Power point- converted to CPD modules for dentists<br>? Is there a guidance booklet.	Documenting the process of development of i/v materials.
Technical Working group/ Purposive and Snowballing.<br>(AB, AMK, KGS, BJK, SJ	Detailed comments on our desk guide and modules<br>Modifications to suit context<br>Materials to include/exclude; how many chapters, etc.	Developed ppt is e-mailed to TWG to get individual feedback first; after corrections a joint meeting to finalise the i/v.<br>The whole process will be documented.<br>Are there any consensus method involved?
6-8 dental practitioners<br>Purposive and Snowballing.	Focus group/ informal discussions with them to find out best ways of implementable record keeping.	Focus group/ informal discussions recorded<br>Results could be incorporated into the intervention and discussed with TWG/ SC.
Steering Committee<br>(IDA Madras Secretary/President)	Discussion to finalise decisions on intervention and endorsement.	
**Feasibility evaluation**		
Around 30 dental practitioners/ Purposive and Snowballing.	Workshop (pilot course) with primary care dental practitioners to refine the intervention.	Evaluated using mixed methods.<br>Can use theory here, if appropriate.

Table S2: Understanding the problem of Inappropriate Antibiotic prescribing in dentistry in India - the 6SQuID approach

QUESTIONS	ANSWERS	
**Nature and extent of main problem**<br><br>What is the nature and extent of problem?<br><br>**Systematic review -**<br><br>**(Bhuvaraghan et al, 2021**	Antibiotic misuse is common in India for routine dental procedures, prophylactically and therapeutically.<br><br>Second/ third line antibiotics and combinations are being used for dental problems.<br><br>Over the counter antibiotic use (self-medication) is prevalent.<br><br>Present at all levels - primary care and teaching hospitals. Prescribed/ dispensed by dentists and non-dentists (GPs, pharmacists, informal healthcare providers) for dental diseases.<br><br>Particularly a problem in primary care setting and outpatient dental care by qualified dental practitioners.	
**Causes and contributing factors**<br><br>What are its possible causes?<br><br>**Policy Document analysis –**<br><br>**Bhuvaraghan et al 2024**	**Levels (Socio-ecological model)**<br><br>**Individual**: **poor knowledge and training, lack of awareness**, compensation for unsure diagnosis, skills, time saving, established habits, knowledge-practice gap, attitude and motivation.<br><br>**Inter-personal**: peer influence, **power balance** (dental student-teacher; dentist-specialist); **dentist-patient communication, perceived patient expectation, patient's habits and oral hygiene.**<br><br>**Institutional**: lack of guidelines, **lack of educational resources**, rules and regulations not enforced, poor infection control practices; lack of resources such as drugs, lab facilities, radiographs.	**Solutions**<br><br>Create/ improve awareness about AMR, CPDs to improve knowledge, read and reflect, record keeping, improve skills & sterilisation, evidence-based practice.<br><br>Communication with patients, educate patients, educate dental team, understanding limitations and appropriate referral, Mentorship.<br><br>Local guidelines and regulations (antibiotic/ IPC), CPDs that are easily available and scaled up.availability of resources, local audits.

	**Community**: dentistry treated as business, **pharma influence**, **prescribing norms, poor community awareness about AMR**, dental problems considered minor by patient, self-medication and OTC antibiotic availability<br><br>**Policy level:** Lack of policies and active prescribing guidelines, poor emphasis in training and curriculum. policy-implementation gap.	Antibiotic and AMR awareness in the community, understanding professional role within community and not yielding to unethical pressure, changing community norms through education and evidence-based practice.<br><br>Developing policies and guidances, curricular modifications. Implementing existing policies on OTC antibiotic use, infection control, record keeping, etc. Audits, supervision and monitoring.
**Consequences**<br><br>What are the consequences for those directly affected?<br><br>What are the consequences for those indirectly affected?	Patient safety issues, cost, prolongation of definitive Rx due multiple antibiotic doses, adverse reactions including allergies and Clostridium difficile colitis, contribution to AMR.<br><br>Community spread of AMR. Burden on health services. Loss of lives, Loss to economy.	

Table S3: Themes on recording prescription data in primary care

Themes	Sub-themes	Quotes
**Recording prescriptions not a norm**	Habitual	No, I don't save it. I just give it to them. FG 5<br><br>We also give it to pt and do not record what is prescribed, but of late I am thinking of recording this data in pt case sheets. As of now, I do not save patient prescription copies. FG 3<br><br>I also give prescription to patient; do not have a record of what I prescribed. Not in case sheet also. FG 6<br><br>if the patient is allergic to a certain medication, I mention that in the case sheet. But I do not routinely record what I prescribed. FG 8<br><br>Nowadays, software can print prescriptions, so we can store a copy if we want. However, with or without the software, I have not been storing prescription data so far. FG 2<br><br>I also write a prescription and give to the patient. Sometimes they bring it back, sometimes they don't. FG 8<br><br>I write a prescription, scan and store it. It is a good practice, actually. But majority of us do not. If you want a change, it must be implementable. FG 1<br><br>Even in the case sheet, I write… 'prescribed Abs and analgesics for ---". Do not write the Ab name and regimen. I would write in the next visit if it responded well. FG 2<br><br>…it would only have been something I studied in the books but not put to use. Unfortunately, I must admit I do not practice what I preach… What is practically possible, only that I do… FG5
	Reliance on patient	Sometimes the pt brings the prescription, but sometimes they have lost it or left it at home, and we won't know (what we prescribed). FG 5<br><br>Sometimes they bring it back, sometimes they don't. FG 8<br><br>Usually, they bring it back. We tell them to bring back all previous records every time they come. FG 2

	Relying on memory	Commonly we prescribe from some 4 different types of Abs only… But difficult to exactly recall what I prescribed. FG 2
	Poor implementation and lack of trust in regulatory body	It may be written somewhere in the Dental council website. But what has the DCI done so far, like this… It will soon be abolished, and Dental commission will take over. Not sure what they will do. I have studied during training, but now, what is practically possible, only that I do. At least, there must be a precedence or there must be forced implementation. FG 5
	Additional work with no perceived benefit	There is no big use to me recording that data now. If I am affected… for e.g. say if I was affected… in the sense some 2 of my patients sued me and I had to go to the court of law because of that, then I will automatically start recording, even without anyone's advice. I have never had such an experience, so I am taking it for granted, I'm not saving it. I have also not heard of anyone around me facing any problems because of this. FG 5<br><br>You all have a big practice and have been practicing for a long time. I have only recently started my practice… FG 6<br><br>For me personally, even feeding it regularly (in the computer) will be difficult. FG 7
**Facilitating adoption of prescription recording practices.**	Penalising/ regulatory enforcement	Imposing fine for non-recording (non-compliance) is the only way to make dentists do this. i.e. By force that during establishing clinic, this should be done. FG 6<br><br>Unless there is force/ disciplinary action, no one would… I myself will not do this. Definitely won't. FG5<br><br>At least there must be a precedence or there must be force/ discipline. FG 5<br><br>Unless, like when clinical registration act came into effect, everyone started registering their clinics; similarly, biomedical waste regulations came… we started segregating wastes. I then started studying about it (waste segregation). Now… even if that person does not come regularly to collect waste, we are used to segregating it because that was brought as a sort of force/ discipline… Otherwise, it would only have been something I studied in the books but not put to use. FG 5<br><br>What I think is we need a little bit of imposed discipline ... Indemnity should become tougher. In another 5 years, everyone is going to need indemnity; at that point, they must put forth conditions such as …. "These data must be recorded for your indemnity to be valid". FG 2<br><br>For example… conditions in their indemnity, pointing to them the NDC guideline if any... it will be somewhere. The NMC has put forth some guidelines for medical practitioners. Not long before dentists get it. FG 2

		For the coming generation of dental trainees (students), it must be mandatorily taught during training in colleges. That is important. If you make this a habit in the institutions… for e.g., using carbon copy prescriptions, asking students to do a survey of antibiotics, then students will understand the importance. FG 2
	Awareness with motivation	Actually, if we want them to keep a record, first, we need to tell them what will happen if they do not do it- it can a be moral or legal reason... FG 2<br><br>Basically, when the patient comes back, you are not embarrassed, you do not need to ask him for previous prescription, because you have a record. FG 2<br><br>To know if this particular antibiotic has worked in this condition. Yes, this is what dentists want to know… self-evaluating your Rx modalities- and refine your Rx modalities. FG 2<br><br>Start building up the reasons and benefits that will make him do it. It is like buying a 2bed apartment that has 'so many' amenities; he might not use any of those facilities but will buy the apartment based on those amenities. FG 2<br><br>See… it'll improve your practice. If you just ask your patient… ' I gave you this medicine last time, did it work, was everything okay?',… patient will be happy that you remembered. FG 2<br><br>Sometimes dentists say that patients are not coming back. The reason is… we are the reason patients are not coming back. We do not know what is happening to the patient. Simple allergy… patient will go to another doctor. So, retain as many records…, patient will be retained. Otherwise, patient will go to another doctor. It is only up to us to retain our patients. FG1
	Practicability and suitability	Moreover, if they have a software, you can tell them, "See you have it all, you just have to stack up a template, and then, a click is going to print and automatically save it. So, please start using your software'. FG 2<br><br>That depends on what suits them. 'If you do not have a software, find out a way that is convenient to you, but please keep a record.' FG 2<br><br>You need not have a big server, big set up, nothing. What you want is a mindset to do it, that's all. FG2<br><br>At least there must be a precedence… FG 5
	Creating fear	We need to give them a couple of alarming reasons for why this is required. If you do so, they'll start working on it. You can tell them, 'this is going to change tomorrow. NDC has taken over from the DCI, it's going to be strict…' FG 2

		They should have some seriousness about their practice. Many dentists feel “I have not faced a problem, so I do not need it”. We should tell them they will face it. FG1<br><br>Legally protecting yourself is very important in this process. Suppose someone puts a case against you… it is not very common now as in other countries, but things are changing rapidly in India. FG 1
**Suggested ways for recording prescriptions**	Recording on tab	I have a tab. It is something like Samsung notes. On it, I have entered case numbers from 1 to 100 for now on excel sheet. I take photos of individual case sheets and add them on my device, for e.g., I have photos of case sheet, patient prescription. I also have manual document. I can retrieve cases by entering patient mobile number on the excel sheet. From this I can identify case number and go to the corresponding case file in my tab/ Samsung notes. This file has all pt details, treatments, pre and post op patient photos, x-rays (rvg)… all these have been saved. FG 7
	Mobile Apps	If there is an app, we can feed it in every day. FG 6
	Photograph	Even feeding it regularly will be difficult. Just taking a photograph is easier. FG 7
	Carbon copy	For me, if we are to store prescription manually, keeping a carbon copy is better. Makes things easier and less time consuming as there is no additional work. FG 3<br><br>If there is carbon copy below the prescription itself, it is very convenient. Scanning may not suit everyone. FG 7<br><br>There are 2 ways: if you want a manual copy, we could have a carbon copy of prescriptions. If you prefer digital, we can do as you suggest (adobe scanner) … either way is okay. FG 3
	Adobe scanner	Best way is to download adobe scanner in your mobile and scan it, then you have a record of everything… I print it (the prescription), I do not write the prescription. FG 1
	Simple table	A simple table also serves the purpose… either in a notebook or computer, where the data is entered and saved. FG 4
	Case sheet	I prefer recording prescription in the case sheet itself where we write patient’s Rx plan… FG 2<br><br>Of late, I am thinking of recording this data in pt case sheets. You may not have a collective data (for retrieval) in the way you want, but you’ll still get it for the individual patient. FG 8

	Digital is better	Digital only going forward... Manual will be difficult. FG 6
**Concerns about using softwares**	Confidentiality	I was using xyz (software), but at that time data safety was an issue, so we couldn't enter much into that. FG 2<br><br>What all data we enter, I still have dilemma in that because our data may not be safe, it goes to a cloud storage, and I don't know what happens to the software. FG 2<br><br>These softwares do not stick to administration; but slowly start promoting you online i.e., making money in promotions…, to makes your online presence big. So you have to pay 'so much' money; they start creating competition with your neighbours. FG 2<br><br>We were also at that time not convinced about software data safety and cloud storage. But in future, things may change. FG 3<br><br>I have patient case sheets and consent form plus xyz software. I just have a subscription for 5 years… Actually, it is good enough in the sense that… from that, we are getting patients…Everyone says that patient identity is a problem. FG 6<br><br>That's why I didn't go for a dental software. Not required for the little data I have. FG5<br><br>I knew they were not transparent. The idea of xyz is not to serve you but make money. FG 1<br><br>xyz did a digital revolution, but of course for their own agenda. In digital record keeping, they have played an important role. Their agenda may be different. However, after its arrival, recording has become convenient and comprehensive also. It covers almost all areas. Other softwares have now come up using xyz as a prototype. FG 2<br><br>I worked in xx community hospital… We used to get crores and crores of patients. But they did not digitize; the reasons they said were 1. You need manual records for court of law; 2. Patient safety (data privacy). FG 7
	Storage	I would wish for a proper offline software which is developed by somebody who understands our dental fraternity… this will be of great use to enter the complete data. But again, storage will be a problem. FG 2
	Time consuming	(I complete) manual records first. Then enter the data either that day, next day or within the week into the software. We don't enter the data every day. FG 5

		Where there are a lot of patients, it is a burden to enter all data at once. We need to allocate time or… a person for this. FG 8<br><br>For me personally, even feeding it regularly (in the computer) will be difficult. FG 7

Table S4: Themes on Chairside antibiotic guide

Themes	Sub-themes	Quotes
**Desk guide format**		I think this is enough, nothing more is required. This will work. FG 1<br><br>It is fine. FG 2<br><br>The guide just needs dose for paediatric patients. FG 6<br><br>The format looks okay. Can understand. FG3 (FG 4,5 agree)<br><br>It looks good. Everything is there. FG 7,8
**Antibiotics prescribing is the norm for various dental conditions**	**Antibiotics considered an essential routine in dentistry**	So, no antibiotics for extractions?! 3,4<br><br>Then… where only do you give Abs?! FG 6<br><br>You cannot skip Abs for any form of abscess, whether it is for localized or periodontal abscess. FG 5<br><br>Yes, you cannot skip (for abscess). Extraction also you cannot skip, as it causes wound, does it not? I had a bad experience… had my impacted 8 removed, and surgeon did not give Abs. I got unbearable pain after 3 days. FG 4<br><br>What if it is a severely infected tooth… you establish drainage and extract the tooth. Everyone's immunity s different. How do you judge here? Or do you by default decide that Abs are not required. FG 2<br><br>I give Abs if the tooth is mobile (gum disease) and I have to extract it. FG 8<br><br>I think gum diseases need antibiotic prescription. I give them antibiotics, then do the procedure. They should not have pain, that's the aim. FG 8
	**Need to compensate for sterilisation**	All instruments we use are not 100% sterile. In surgical OT, we use surgical gloves. We only use normal gloves for surgical procedures that are not sterile. FG 7

		If you take endodontic treatment (RCT), suppose you call the endodontist for 5 patients, he uses one file only, wipes it and reuses for all patients… I am talking about what actually happens in practice… (everyone laughs) FG 6<br><br>No, they (endodontists)are not doing (sterilizing) it. FG 6<br><br>Sterilisation is mandatory. You cannot go in that line. FG 2
	**Doctor's peace of mind/ just in case**	Here, we give Abs mainly for doctor's peace of mind. FG 2, 3, 6.<br><br>"Whatever may happen, Abs will take care of it." FG 2<br><br>If I do an extraction or impaction and patient develops fever and swelling next day, then I have to call the patient and write a prescription… That's why we write Abs in advance. FG 6
	**Gaining patients' respect and goodwill/ Fear of losing patient**	In the first place, if you didn't write a prescription, pt will ask if you are indeed a doctor and if he can see your certificate. This has actually happened to me. FG 5<br><br>In simple terms… It is a matter of survival. With patients, it has become a service industry more than a medical field. "You go to the doctor; your problem gets solved in one day. With minimal expenditure … then you are good. FG 2<br>My clinic is in a closed residential community. Information spreads by word of mouth. If my patient has one problem, my clinic will be empty for the next week FG 6<br><br>And if we are to make files (endodontic) disposable/ single use only, we need to increase RCT fees. FG 5<br><br>Basically, dentists are scared patients will not come to them if they do not prescribe. FG8
	**Problems of fresh graduates**	These dentists (pointing to FG1 and FG2) are already established. The problem is… patients listen to doctors because you are already an established practice. that's not the case with us. FG 5<br><br>When you are an established practitioner, we can do according to what we want; In the past, we learnt as we treated patients; now you must learn everything and then only come in. There are 2 aspects- one is getting established, the other is earning… if you have earned enough to sustain, you can take a stand… But it takes a bit of time... That's what I say… You talk about doing good to patients. Here, patients are fine, it is the dentists who are suffering… FG 3

	**Individual dentist's Attitude**<br><br>**Dentist's beliefs**	Nowadays patients ask for the charge of treatment and then only come to us. We are forced to think… we'll live with whatever we earn, pay rent, and carry on with our lives. FG 6<br><br>After all this this talk here, if I get 10 patients tonight, I am going to have to prescribe Abs for all 10 pts. Whether they need it or not. I am telling practically. FG 5<br><br>I do lot of procedures under i.v. sedation. I have noticed that healing and recovery process is very fast, I do not know if it is something to do with happy hormones or reduced anxiety. Because, we just give midazolam, nothing else, no antibiotics. Another thing to explore. FG 1<br><br>Vegetarian patients in my observation do not tolerate antibiotics well… may be something to do with gut behaviour), so I avoid as much as possible. FG 1
	**Antibiotic of choice**	This antibiotic alone (points to amoxy), I am not comfortable with this. Maybe you could put… '*antibiotic of your choice*' here… FG 1<br><br>As a practice, we give Amoxy with clavulanic acid only. Even in that, some brands do not work. FG2<br><br>Plain amoxy does not work- Only with clavulanic acid, it works. Otherwise, I give cephalexin. FG1<br><br>I use injectable Abs regularly for pts with e/o swelling. I also give i.m. Abs before surgical procedures. FG 5<br><br>I don't think amoxy works. In case I have to give Abs, I give only Amoxiclav. FG 1<br><br>Now after covid, azithromycin has become resistant. We are using more of Clindamycin. FG 4<br><br>They keep changing their prescribing pattern. FG 3<br><br>They prescribe the same combinations again and again, and when something new comes, they change to it. FG 4
**Patient communication/ awareness is underestimated**		My point is, when patient comes with any infection, there will be a tendency from the pt side to ask for a prescription. Because they have been treated that way for generations. It is our bounded duty to tell them that they do not need Abs. FG 1<br><br>Many patients do NOT know what Abs are. FG 8

		They know VERY well. He (patient) thinks if he gets an Ab, he need not pay for RCT. From the perspective of those who demand Abs, 'dentists are doing RCT to make money to treat a condition that can be cured by antibiotics. So, why do I have to undergo a procedure when medicines can cure my problem.' FG 1<br><br>Only a patient who wants to leave you will ask for Abs; if he trusts you, he will stay and listen to you. FG 1<br><br>My employee once asked me 'Why are you talking so much to the patient? You just tell them and do the procedure'. FG 6
**Procedure essential**		I tell my patients… "Look… I am the surgeon. I will work on you and take care of your pain. You don't need an Ab. I am your Ab". Take a bp blade and handpiece. If these two things are there in your practice, 90% of Abs usage can be stopped. If you are ready to take blade and hand piece… FG 1
**Managing sales representatives**		What I learnt is… many young dentists- first 5-10 years of practice…They see lot of (sales) representatives… this rep meeting should be banned. For some reason that a person comes regularly and begs… some dentists feel sorry (for them) and prescribe. I don't entertain any medical reps. If anyone comes, we politely say 'we don't write what you say, so please don't wait here'. Already, we use only 2-3 antibiotics that we use as standard; this culture of somebody coming and telling you what we need to prescribe, and patients asking for Abs should be revisited. FG 1
**Dentist awareness and education**		Ideally, we need to go to every clinic and talk to dentists. FG 1<br><br>The first reason why dentists have poor practice is because of antibiotics. Yes, our practice gets affected. Patients don't go for Rx at all. FG 2<br><br>My personal opinion is that AMR is NEVER going to be caused by dentists in this lifetime. Dentists can never be a cause for AMR. We use very limited number of antibiotics. Mostly amoxy. How can just amoxy lead to AMR? FG 5<br><br>IDA or someone should take the baton and make dentists aware. Awareness must happen like a 'movement' basically. FG1
**Government not doing enough**		During covid time, they were able to successfully implement 'No OTC sale'. That means it is possible, isn't it?<br>Certainly… it can be done if they are determined. FG 5

Table S5: Outline of the final intervention (training module)

1. Objectives of the Training module
2. Antimicrobial Resistance – what is it?<br>• Dental antibiotic use and drug resistance<br>• Spread of resistance<br>• Video – on AMR by expert<br>• Why AMR matters – Global statistics<br>• Key terms about antibiotics<br>• India's resistance scenario and statistics<br>• Video – from Researcher
3. Antibiotic Stewardship
4. Antibiotics in Dentistry, and misuse<br>• Video – dental stakeholder<br>• Interactive question and answers on AMR<br>• Indications for Antibiotics in Dentistry<br>• Chairside Antibiotic Guide<br>• Summary table – when to and when not to prescribe therapeutic antibiotics in dentistry.<br>• Common antibiotics used in dentistry<br>• Dental Pain management – pain ladder<br>• General Principles of Antibiotic prescribing in dentistry<br>• Self-Assessment case scenarios<br>• Prophylactic antibiotic prescribing<br>• Common non-clinical reasons - Patient safety and avoiding unnecessary prescribing<br>• Self-assessment question and answers on antibiotic prescribing

5. Recording prescribing – why is it important<br>• Legal Requirements in India on record keeping in dentistry<br>• Ways of recording prescription data
6. Involving Patients<br>• Why is communication important<br>• Case scenario<br>• Sample patient information sheet for communication
7. References

Table S6: The TIDieR (Template for Intervention Description and Replication) Checklist Information to include when describing an intervention and the location of the information

Item number	Item	Where located **	
		Page number in the manuscript	Other † (details)
	**BRIEF NAME**		
**1.**	Provide the name or a phrase that describes the intervention.	1	A dental antibiotic stewardship intervention for India
	**WHY**		
**2.**	Describe any rationale, theory, or goal of the elements essential to the intervention.	2,3	This stewardship intervention will help improve awareness about antimicrobial resistance and increase knowledge about dental antibiotic prescribing guidelines among dental practitioners, leading to improved prescribing behaviour (reduce misuse) for dental diseases and conditions.
	**WHAT**		
**3.**	Materials: Describe any physical or informational materials used in the intervention, including those provided to participants or used in intervention delivery or in training of intervention providers. Provide information on where the materials can be accessed (e.g. online appendix, URL).	8-9	The intervention included a training module, a chairside antibiotic guide and a patient information sheet. The training module was to be delivered in a power point format. After the training, each practitioner gains access to a copy of chairside antibiotic guide to use in their practices, along with a patient information sheet to enable better communication with their patients.

**4.**	Procedures: Describe each of the procedures, activities, and/or processes used in the intervention, including any enabling or support activities.	9	The training, in the form of power point module, was self-paced and designed to be undertaken individually by participant dental practitioners on their laptop computers. The module and associated materials were developed and refined by dental practitioners and stakeholders from India to make it acceptable to local context.
**5.**	**WHO PROVIDED**<br>For each category of intervention provider (e.g. psychologist, nursing assistant), describe their expertise, background and any specific training given.	9	This was a self-paced training intended to be converted to an online CPD-type resource for dental practitioners to access on their own. No specific training was necessary to provide the intervention.
**6.**	**HOW**<br>Describe the modes of delivery (e.g. face-to-face or by some other mechanism, such as internet or telephone) of the intervention and whether it was provided individually or in a group.	9	The intervention was developed in such a way as to enable dental practitioners to access online using their gadgets.
**7.**	**WHERE**<br>Describe the type(s) of location(s) where the intervention occurred/ intended to occur, including any necessary infrastructure or relevant features.	9	To be undertaken by dental practitioners online using their gadgets at their convenience of home/ clinic/ office.

8.	**WHEN and HOW MUCH**<br>Describe the number of times the intervention was delivered and over what period of time including the number of sessions, their schedule, and their duration, intensity or dose.	9	Dentist to undergo the training (reading the module) once depending on available time and understanding; It takes approximately 40 min to an hour to read the module once.
9.	**TAILORING**<br>If the intervention was planned to be personalised, titrated or adapted, then describe what, why, when, and how.	-	n/a
10.	**MODIFICATIONS**<br>If the intervention was modified during the course of the study, describe the changes (what, why, when, and how).	6-16	The intervention was refined with stakeholders through an iterative approach as part of development process.
11.	**HOW WELL**<br>Planned: If intervention adherence or fidelity was assessed, describe how and by whom, and if any strategies were used to maintain or improve fidelity, describe them.	9	Dentists will complete the content of the module once within the specified time (max 1 hour) on their personal devices. Engagement with the intervention will be monitored. No interactions will be made with participants during the training apart from sorting out technical glitches. All participants will gain access to the chairside guide and patient information sheet on completing the training.
12.	Actual: If intervention adherence or fidelity was assessed, describe the extent to which the intervention was delivered as planned.		n/a

Table S7: GUIDED (Guidance for reporting Intervention Development studies in health research) Checklist. A guideline for reporting for intervention development studies.

Item description	Explanation	Page in manuscript where item is located
Report the context for which the intervention was developed.	The intervention was co-developed by researchers with dental stakeholders from Chennai, India, for primary care dentists practising in India.	2,3,5,6
Report the purpose of the intervention development process	The intervention was developed with the objective of reducing inappropriate prescribing behaviour among dentists in India, identified in a recent systematic review performed by the authors, and the lack of CPD programmes identified in a policy document analysis.	2,3
Report the target population for the intervention development process	The primary target of the intervention is primary care dental practitioners in India, most of whom work in private practice. Since the intervention is designed to be delivered online, it can be accessed by dentists across diverse demographics and geographical locations throughout the country. This ensures equitable access to the intervention and has the potential to benefit patients and the wider public nationwide.	3
Report how any published intervention development approach contributed to the development process	The development of the current intervention was guided by the MRC framework for development of complex interventions. We have also used the 6SQuID approach (supplementary table 2) to clarify the research problem, understand the causes, identify the area to intervene. The development followed an 'embedded development and research' approach (Walley J 2019) wherein the intervention was co-produced with local stakeholders considering local resources and needs and designed from the outset for scale-up.	Pages 3-7 and supplementary table 2
Report how evidence from different sources informed the intervention development process.	Evidence from systematic review and a subsequent policy document analysis demonstrated the clinical/ non-clinical areas of inappropriate antibiotic prescribing by dental practitioners in India and highlighted the need for educational resources for dentists. Prior success by members of research team in developing and evaluating online educational resources in LMIC settings also informed the development process. The intervention also draws on global guidelines and evidence on antibiotic prescribing in dentistry.	8-10

Report how/if published theory informed the intervention development process.	We used the programme theory to clarify factors underpinning the intervention. Models that aligned with the causal assumptions and mechanisms of programme theory were also considered such as the COM-B and socio-ecological model, although we refrained from imposing a single established theoretical framework during the development stage.	5
Report any use of components from an existing intervention in the current intervention development process	n/a	
Report any guiding principles, people or factors that were prioritised when making decisions during the intervention development process.	The development was guided by the principles of implementation science, i.e., all attempts were made to ensure that the intervention was implementable in the target population, and was sustainable and scalable, so we followed a co-production and embedded approach. The contents and format were therefore prioritized based on advice from stakeholders considering local needs. Furthermore, the online mode offered affordability, practicability and equity.	6-9
Report how stakeholders contributed to the intervention development process	Stakeholders were involved even before the start of the development process, in a previous study where they helped identify relevant dental documents. During the development process, they were involved at different stages, such as with planning, identifying participants, designing the contents and format of the resources, endorsement and dissemination.	3,4, Table 1
Report how the intervention changed in content and format from the start of the intervention development process.	As informed by small exploratory research (focus group discussion) during the development process, it became necessary to include record keeping and prescription recording in the training module. This was the most substantial change. In addition, due to the iterative nature of the process, other changes to format and content were made based on stakeholder inputs as described in the manuscript.	11-15
Report any changes to interventions required or likely to be required for subgroups.	There is potential to adapt the intervention to dental students, and diverse contexts in India with considerations to local culture, beliefs and language. There is also potential to adapt the intervention to similar LMIC contexts.	18

Report important uncertainties at the end of the intervention development process	At the end of the development process, certain uncertainties remained, concerning prescription recording practices of dental practitioners, perception of the intervention and format, and utility of various intervention tools.	18
Follow TIDieR guidance when describing the developed intervention	TIDieR checklist has been used (please see appendix 8) to describe the intervention.	Supplementary table 6
Report the intervention development process in an open access format.	This study will be published in an open access format to ensure the development process is accessible to all.	n/a

Figure S1: Draft Logic model of dental antibiotic stewardship intervention

Situation
Inappropriate dental antibiotic prescribing in terms of indications, and antibiotic choice and regimen. No prescribing guidelines and poor dental antibiotic stewardship programmes in India.

Evidence synthesis
Quantitative and qualitative- systematic review and 'policy document' analysis.

To consider
Local dynamics and context (dentist perception and beliefs, resources, etc. Co-production with stakeholders.

Core elements
- Awareness about AMR and professional commitment.
- Diagnosis of dental diseases and knowledge on when to prescribe.
- Reinforcement of appropriate Ab use (avoid combination and high-end Abs and prefer narrow spectrum alternatives.
- Recognising strengths and limitations and act for patient's best interests.
- Preventive measures (diet/Oral hygiene/ habits)
- Patient education

INPUTS

***STAFF/ WORKFORCE**
1. Steering Committee (Policy personnel, persons from the IDA and DCI) – in managing and disseminating resources.
2. Technical Working Group (professionals and other personnel with knowledge and interest in AMR – infectious disease, microbiology, and dental personnel– resource production and delivery
3. Volunteers – in co-production and refinement of resources.

* **TIME** to produce resources, conduct training.

* **FINANCES** (ensuring cost-effectiveness), preparing online module, arranging for workshop, feasibility assessment.

***TECHNOLOGY AND PRODUCTS** (e.g., computers, recording devices, monitoring programmes, drives, sample prescriptions, IT

*** RESEARCH SETTING/ WORKSPACE**

ACTIVITIES

TWG creates resources for dental practitioners, trainees, and patients

IT personnel to convert this into online CPD module usable by practitioners

Workshop with a sample of 15 dental practitioners in Chennai to pre-test (pilot) materials which included:
a) training for 15 dentists in AMR awareness, dental Ab prescribing, record keeping, patient communication and other stewardship areas.
b) Refining the developed training materials, guidances.
c) Interviews/ discussion on feasible and acceptable ways implementing dental Ab Stewardship (barriers/ facilitators)

Steering Committee disseminates/ endorses resources.

OUTPUTS

*A one-page job aide for quick reference
*CPD Module on AMR and Ab prescribing developed for dental practitioners and trainees.
*Pt education material (leaflets).

* 15 dental practitioners trained on AMR awareness and dental Ab prescribing.

*Developed intervention package refined.

*Feasible and acceptable ways of record keeping; barriers and facilitators to implementation.

*CPD Module to be available on Regional Dental Association website.

OUTCOMES
Processes

Improved knowledge and awareness on AMR and commitment to tackling it.

Attitude changes about prescribing.

Ability to diagnose if a dental condition does/ does not require antibiotics.

Practice change- Improved Ab prescribing.

Understanding limitations & prompt referral by dental practitioners, Better Rx / patient safety

Improved communication with patients leading to patient awareness about AMR

Ability to record prescribing data- record cards

Policy created for primary care dental service

IMPACT

Reduction in dental burden of AMR

Improved Patient safety and reduced complications

Patient understanding of AMR leading to reduced self-medication

Surveillance data made available

Standardisation of dental antibiotic prescribing

Figure S2: Topic guide for Focus Group Discussions

An Intervention to reduce inappropriate antibiotic prescribing among dental practitioners in India

<u>Recording</u>

Do you record patient data? Who records?

What data do you record for each patient?

General data: name, age, etc.

Patient-specific data: patient's chief complaint, diagnosis, other findings, consent forms, photographs, prescriptions, any follow-up, etc.

How do you record patient-specific data?

Do you record your prescribing? How?

How is this data stored and retrieved when required?

<u>Legal requirement</u>

Do you know if any law exists from the MCI/ DCI or government, on whether patient data need to be recorded stored, and for how long this needs to be stored?

<u>How to make it work?</u>

Can you suggest ways to improve/ introduce and maintain record keeping?

<u>Self-evaluation</u>

If you are regular in recording, have you ever self-evaluated your prescriptions to improve prescribing pattern?

How appropriate do you think your prescriptions are and Factors affecting prescribing.

Do you think a little help in this regard will be useful?

<u>Comment on desk guide</u>

Could you comment on this one-page document? It is called a 'chairside desk guide' and helps dentists whenever they are in a dilemma on whether the patient needs antibiotics.

You could also write down your comments on the document, for e.g., regarding referral for cardiac opinion, communication with patient, etc.
